# Association between perceived environmental pollution and health among urban and rural residents-a Chinese national study

**DOI:** 10.1186/s12889-020-8204-0

**Published:** 2020-02-06

**Authors:** Ting Yang

**Affiliations:** School of Sociology, Huazhong University of Science & Technology, 1037 Luoyu Road, Wuchang, Wuhan, 430074 China

**Keywords:** Perceived environmental pollution, Self-rated health, Urban residents, Rural residents, China

## Abstract

**Background:**

China’s economic boom has led to severe environmental pollution, which has created significant health risks for residents. Although current studies have found urban residents can sense the harmful effects of environmental pollution in China, few studies have talked about their rural counterparts’ attitudes towards the health impacts of environmental pollution. Similarly, little research has talked about the inequality of environmental awareness between urban and rural residents.

**Methods:**

Descriptive and analytical statistics were used for the data analyses based on a national survey, namely, T*he 3rd Survey on the Status of Chinese Women in 2010*, which was jointly conducted by the All China Women’s Federation and the China Statistical Bureau in 2010. A total of 24741observations were selected.

**Results:**

Among urban residents, 67.21% reported that their total health was good, which was 1.35% lower than the reported rate of their rural counterparts; 25.88% of urban residents reported that their total health was general, which was nearly 3% higher than the reported rate of their rural counterparts; 6.91% of urban residents reported that their total health was poor, which was 1.63% lower than the reported rate of their rural counterparts. The study also found that the rates of urban residents who perceived air pollution (35.67%), water pollution (17.96%), garbage pollution (25.05%), and noise pollution (32.05%) were higher than those of their rural counterparts. Perceived air pollution, and perceived noise pollution both had a negative effect on urban residents’ good health (B = − 0.14, *p* < 0.05; B = -0.23, *p* < 0.001). Perceived garbage pollution had a positive effect on urban residents’ poor health (B = 0.33, *p* < 0.01). Perceived water pollution had no significant effect on urban residents’ health. The four types of perceived environmental pollution all had insignificant effects on rural residents’ health.

**Conclusions:**

Rural residents lack awareness of the impacts of environmental pollution on health, which may create risks and vulnerability within the rural environment and the livelihood of these residents. Great attention should be paid to the impacts of environmental pollution on the health of not only urban residents but also rural residents, which will highly improve the support of green development among the public in China.

## Background

The emergence of a quantity of mega cities accompanying China’s economic boom has led to enormous increases in resource consumption and a variety of pollution. Environmental pollution has become one of China’s top environmental concerns [1]. Air pollutants, such as carbon monoxide (CO), sulfur dioxide (SO_2_), nitrogen oxides (NOx), volatile organic compounds (VOCs), ozone (O_3_), heavy metals, and respirable particulate matter (PM2.5 and PM10), vary in their chemical composition, reaction properties, emission, disintegration time and potential to diffuse over long or short distances [2]. Air pollution has negative effects on a wide variety of human systems and bodies, causing worsening respiratory symptoms, more frequent use of medication, decreased lung function, recurrent use of health care and accelerated mortality [3]. The World Health Organization ranked air pollution as the world’s 13th leading cause of mortality as the pollution from particulate matter (PM) contributes to approximately 800,000 premature deaths every year [3].

Water quality issues are a primary challenge facing humanity in the twenty-first century [4]. The foremost sources of water pollution include chemical pollution, specifically inorganic and organic micropollutants consisting of toxic metals and metalloids as well as a range of synthetic organic chemicals [4]. The quantity of wastewater produced has expanded unexpectedly due to the developing volume of industrial chemicals, heavy metals, and algal toxins, in addition to China’s economic-related increase; this increase in wasterwater quantity has been linked to unfavorable health effects such as deaths from liver and stomach cancer [5]. The Ministry of Water Resources has monitored 532 rivers in China and found that 436 rivers are polluted to varying degrees; among the 15 major urban rivers flowing through the seven major rivers of China (i.e., the Yangtze River, the Yellow River, the Huaihe River, the Pearl River, the Liaohe River, the Haihe River, and the Songhua River), 13 rivers have serious water pollution, accounting for 87% [6]. Rapid, unregulated industrial increase and urbanization without sufficient investments in the water supply have exacerbated waterborne infections and parasitic diseases [7]. Industrial pollution and wastewater are the main sources of water pollution.

Large amounts of household garbage produced due to an overpopulation of residents are piled on the ground, producing bacteria and polluting the air and groundwater. Fertilizers and pesticide residues produced in agricultural production have caused pollution to the soil and groundwater as well. The garbage production in China is the highest worldwide and nonetheless increases at the speed of 8–10% every year [8]. China has tried to enforce the policy of classifying household garbage in the eight cities of Beijing, Shanghai, Guangzhou, Shenzhen, Hangzhou, Nanjing, Xiamen, and Guilin since 2000; however, none of the cities have succeeded due to various factors such as related policies, residents’ attitudes and values [8]. A report published by the Chinese Ministry of Environmental Protection showed in 2015, the domestic garbage production of 246 large and medium-sized cities was 185.64 million tons; the disposal rate and the harmless treatment rate were 97.3% [9], and, 92.5% [10], respectively. Among these cities, the largest amount of household garbage was generated by Beijing, with a production of 7.903 million tons, followed by Shanghai, Chongqing, and Shenzhen [9]. The household garbage production in rural areas amounts to 0.3 billion tons every year, accounting for 75% of the garbage production in urban areas [11]. In addition, China’s township domestic garbage disposal rate and harmless treatment rate have been reported as 50%, and, 13.96% [12], respectively. With insufficient environmental awareness, rural residents messily dump their garbage in a mess, thereby increasing the difficulty related to garbage collection and treatment. This outcome is partly due to insufficient investment in waste pollution technology and lagging legislation and policies [13]. The majority of waste disposal facilities in rural areas have only been built in the last few years. The increasing and unclassified rural household waste has not only polluted the earth but also the air and water.

As an essential public health problem, noise can lead to hearing loss, sleep disruption, cardiovascular disease, social handicaps, reduced productivity, impaired education and learning, absenteeism, accelerated drug use, and accidents [13]. It has often been considered less harmful than other pollutants [15], although noise exposure in rural and urban areas is prevalent in everyday life. Busy traffic with a variety of vehicles both day and night and noisy construction sites can be the offenders against a quiet living environment anywhere [16]. With the strong implementation of the urbanization strategy over the last 10 years, centralized residence in villages and towns has become very common. Meanwhile, road building has also been greatly encouraged [17]. Labor-intensive industries, processing factories and agricultural products enterprisess are emerging in the countryside.

Sustainable development as a basic national strategy has been established since 1992 in China. However, environmental pollution and ecological degradation have continued to increase and have inflicted high levels of damage on the economy and quality of life [14]. China starts from a point of grave pollution and prioritizes progress in the urban environment during its current stages of development. The pressure-based government assessment mechanism is the driving force for transferring environmental pollution from urban to rural areas [15]. Economic growth remains the primary mission of rural development, and industrialization is the foremost way to achieve this goal. Peasants prefer to earn money, and the government pursues political achievements. Under this pressure, villages permit heavily polluting enterprises to produce nearby. Openness, ambiguity and the publicity of the environmental rights of peasants make environmental degradation in rural areas particularly serious [16]. Studies on the harmful effects of objective environmental quality on health have mostly been conducted in urban areas in China. Measures aimed at controlling environmental pollution have been taken mainly in cities. This approach has created an illusion that environmental pollution in Chinese cities is much more serious than that in Chinese villages. Rural residents cannot rationally judge the harm of environmental pollution, as they benefited from the immoderate use of noticeably cheap natural resources and the continuous transfer of environmental pollution due to loose environmental policies in their areas [15, 16]. The lack of studies on the distribution of environmental quality at both urban and rural scales has resulted in little understanding of environmental quality and health inequality.

Moreover, the extant studies have focused on the relationship between the environment and health in the fields of environmental epidemiology and environmental toxicology [17]. The studies have mainly measured objective environmental pollution, such as pollution levels [18] and pollution types [17], and have discussed their relationships with mortality. Although current studies have found urban residents can sense the harmful effects of environmental pollution in China [19], few studies have talked about Chinese rural residents’ attitudes towards the health impacts of environmental pollution. Furthermore, little research has talked about the inequality of environmental awareness between urban and rural residents based on their social and economic perspectives, which has resulted in the lack of pertinence and effectiveness for the corresponding environmental and health policies in China. In addition, China’s governments have recognized that environmental protection, rather than being a drag on the economy, can ensure long-term sustainable economic development. Green development has been named as one of the five key drivers for development in the 13th Five-Year Plan (2016-2020)[20]. However, a very limited number of studies have been conducted to evaluate Chinese residents’ attitudes towards the health impacts of environmental pollution despite the severe objective levels of environmental pollution. Therefore, this study aims to investigate urban and rural residents’ perceived environmental pollution and their harmful effects on health.

## Method

### Data source

The data were from *The 3rd Survey on the Status of Chinese Women in 2010* (SSCW3) jointly conducted by the All China Women’s Federation (ACWF) and the China Statistical Bureau in 2010. The sampling of this survey is conducted in four stages. The first stage of sampling is based on the districts of cities above the prefecture level and the county and county-level cities. The primary sampling units of the three municipalities of Beijing, Tianjin and Shanghai are townships, towns and streets. The second stage of sampling is to draw 5 resident (urban) or village (rural) committees based on the level of urbanization in each of the districts and counties (township streets) that are selected. The sample districts and counties (township streets) and the resident or village committees are selected by the sampling expert group of the National Investigation Leading Group Office according to the sampling plan and sampling frame data. The third stage is sample selection of the households in the resident or village committee, which adopts equal probability sampling of random starting points, that is, equidistant sampling. A fixed number of 15 households are selected for each resident or village committee. The fourth stage is to select samples in the household. Specifically, the number of family members in the household is taken, and the respondents are determined by random selection. The third and fourth stages are conducted by the investigator. The cross-sectional data were collected from nationally representative samples through self-administered surveys. The questionnaires used in the SSCW3 covered a major and an additional questionnaire. All the participants verbally agreed to participate. The total number of samples in the major questionnaire was 26,171 and the response rate was 99.0%. Female and male adults aged 18–65 years with *hukou* information in the major questionnaire were selected. Finally, 24,741 samples were kept.

### Questionnaire

A structured questionnaire was developed and used for the investigation (Additional file 1). The questionnaire was anonymous and measured the participants’ attitudes subjectively. The questionnaire used in this study were part of the primary questionnaire and consisted of the questions: (1) respondents’ general demographic characteristics, (2) perceived environmental pollution, (3) self-rated health. The demographic questions in section 1 included their gender, age, hometown or places they were brought up (rural versus urban), marital status (never married, married, divorced, widowed), education level (primary school or below, junior or senior high school, postsecondary or above), the father’s education level.

The participants were asked four questions about environmental pollution: Did you perceive air/water /garbage /noise pollution in your daily life? (Yes versus No). If the answer was “Yes”, perceived air/water/garbage/noise pollution was defined “1”, otherwise defined “0”. All the variables were binary variables. The participants were also asked their health status measured using a Likert scale with five levels, from very good or good versus general versus poor or very poor. Self-rated health has been shown to be a reliable and valid measure of general health status [28] and has been reported to be associated with various objective health measures and strongly predicts future onset of mortality [29, 30].

### Data analysis

Stata software version 15.0 (Stata Corp., College Station, TX, USA) was utilized for the data examinations. The analyses consisted of three parts. First, descriptive statistics were used to present the demographic characteristics, perceived environmental pollution and health status of the participants. These data were presented as “percentages”.

Second, univariate examinations were performed by cross-tabulations, utilizing Likelihood-ratio Chi-square tests, to determine relationships between perceived air pollution, water pollution, garbage pollution, noise pollution (independent variables) and health status (dependent variables).

Third, multivariate examinations were directed to additionally determine connections between the independent variables and dependent variables. For the dependent variable, health status was set as a categorical variable (0 = general health, 1 = good health, 2 = poor health). Hence, multinomial regression examinations were directed eventually. Perceived environmental pollution’s impacts on health status were assessed for urban and rural residents, respectively. Gender, age, marital status, education, father’s education was controlled. A *p*-value less than 0.05 was defined as statistically significant.

## Results

### Descriptive statistics

A total of 24,741 individuals were analyzed. Among them, approximately half were female (51.29%), and resided in urban areas (46.14%). The majority of them were married (84.03%), completed an education of junior or senior high school (58.28%). Most of their father completed an education of primary school or below (66.0%). In general, the age distribution of 18–29(16.54%), 30–39(24.01%), 40–49(29.51%), 50–59(22.31%), 60–65(7.63%) year-old was balanced. Among the urban residents, 67.21% reported their total health was good, which was 1.35% lower than the reported rate of their rural counterparts; 25.88% of urban residents reported their total health was general, which was nearly 3% higher than the reported rate of their rural counterparts; 6.91% of urban residents reported their total health was poor, which was 1.63% lower than the reported rate of their rural counterparts.

### Demographic characteristics

Table 1 presented the residents’ demographic characteristics. Among the urban residents, 47.94% were male; the rate of their rural counterparts was 49.37%. 15.69% were 29 years or younger, 7.31% were 60 years or older; the rate of their rural counterparts was 17.26, 7.90%, respectively. 8.51% had completed education through primary school or below, more than three fifths (61.20%) had completed education through junior or senior high school, about 30% had completed postsecondary or above; the rate of their rural counterparts was 42.32, 55.78, 1.90%, respectively. 10.86% of urban residents were never married, 81.94% were married; the rate of their rural counterparts was 9.03, 85.82%, respectively. More than half (51.53%) of urban residents’ father had completed primary school or below, 41.56% of their father had completed junior or senior high school, 6.91% of their father had completed postsecondary or above; the rate of their rural counterparts was 78.39, 21.23, 0.38%, respectively.

**Table 1:** Demographic characteristics of residents (*N* = 24,741)

Variable	Urban residents(*N* = 11,416)					Rural residents(*N* = 13,325)					p-value
		Good	General	Poor	p-value		Good	General	Poor	p-value	
Total		67.21	25.88	6.91			68.56	22.90	8.54		***
Gender											
Male	47.94	71.37	22.58	6.05	***	49.37	72.77	20.42	6.81	***	*
Female	52.06	63.39	28.91	7.71		50.63	64.44	25.33	10.23		
Age											
≤ 29 years	15.69	87.72	11.22	1.06	***	17.26	87.83	10.48	1.70	***	***
30–39 years	25.23	75.66	20.58	3.78		22.97	77.95	18.03	4.02		
40–49 years	29.67	63.09	27.34	9.57		29.37	67.35	24.55	8.10		
50–59 years	22.10	53.82	36.46	9.71		22.49	54.92	30.53	14.55		
≥ 60 years	7.31	51.26	37.72	11.02		7.90	42.45	36.37	21.18		
Education level											
Primary school or below	8.51	48.30	35.63	16.07	***	42.32	57.76	28.04	14.20	***	***
Junior or senior high school	61.20	64.78	27.31	7.91		55.78	76.19	19.36	4.45		
Postsecondary or above	30.29	77.44	20.24	2.31		1.90	84.98	12.65	2.37		
Marital status											
Never married	10.86	85.89	10.89	3.23	***	9.03	82.38	12.47	5.15	***	***
Married	81.94	66.26	27.11	6.63		85.82	68.19	23.45	8.36		
Divorced	4.21	54.89	30.98	14.14		1.45	63.21	27.46	9.33		
Widowed	2.99	42.82	39.30	17.89		3.71	45.34	34.01	20.65		
Father’s education level											
Primary school or below	51.53	60.77	30.02	9.21	***	78.39	65.60	24.73	9.67	***	***
Junior or senior high school	41.56	74.14	21.27	4.60		21.23	79.32	16.26	4.42		
Postsecondary or above	6.91	73.64	22.69	3.68		0.38	76.00	18.00	6.00		

^*^ *p* < 0.05, ^**^ *p* < 0.01, ^***^ *p* < 0.001

### Perceived environmental pollution

Table 2 presents the summary of residents’ perceived environmental pollution. 35.67% of urban residents perceived air pollution in their daily life, 18.91% higher than the rate of their rural counterparts; 17.96% of them perceived water pollution, 5.5% higher than the rate of their rural counterparts; 25.05% perceived garbage pollution, 6.86% higher than the rate of their rural counterparts; 32.05% perceived noise pollution, 18.56% higher than the rate of their rural counterparts.

**Table 2:** Perceived environmental pollution among urban and rural residents

Variable	Urban residents (*n* = 11,416)					Rural residents (*n* = 13,325)					p-value
		Good	General	Poor	P-value		Good	General	Poor	p-value	
Air pollution											
Yes	35.67	62.84	29.17	7.98	***	16.76	69.10	24.23	6.67	***	***
No	64.33	69.64	24.05	6.32		83.24	68.45	22.64	8.92		
Water pollution											
Yes	17.96	61.32	30.63	8.05	***	12.46	67.23	25.18	7.59	*	***
No	82.04	68.50	24.83	6.66		87.54	68.74	22.58	8.68		
Garbage pollution											
Yes	25.05	61.40	29.48	9.13	***	18.19	68.19	24.71	7.10	**	***
No	74.95	69.16	24.67	6.17		81.81	68.64	22.50	8.86		
Noise pollution											
Yes	32.05	62.18	30.01	7.82	***	13.49	68.21	22.87	8.93	***	***
No	67.95	69.59	23.93	6.48		86.51	70.78	23.15	6.07		

^*^ *p* < 0.05, ^**^ *p* < 0.01, ^***^ *p* < 0.001

### Inferential statistic

All the factors associated with health among urban and rural residents were displayed in Table 3 respectively.

**Table 3:** Multinomial regression models of perceived environmental pollution’s impacts on health

	Urban residents		Rural residents	
	Model 1a	Model 1b	Model 2a	Model 2b
	Good vs General	Poor vs General	Good vs General	Poor vs General
	B(95%CI)	B(95%CI)	B(95%CI)	B(95%CI)
Air pollution (No)				
Yes	-0.14^*^ (− 0.26,-0.02)	0.09 (− 0.12,0.31)	− 0.12 (− 0.26,0.02)	−0.11 (− 0.35,0.14)
Water pollution (No)				
Yes	− 0.10 (− 0.25,0.04)	− 0.18 (− 0.42,0.07)	−0.06 (− 0.22,0.09)	−0.03 (− 0.28,0.23)
Garbage pollution (No)				
Yes	−0.04 (− 0.18,0.09)	0.33^**^ (0.10,0.56)	− 0.08 (− 0.21,0.05)	−0.17 (− 0.40,0.06)
Noise pollution (No)				
Yes	−0.23^***^ (− 0.35,-0.11)	−0.19 (− 0.40,0.03)	−0.06 (− 0.21,0.09)	−0.10 (− 0.36,0.16)
Gender (male)				
Female	−0.38^***^ (− 0.47,-0.29)	−0.08 (− 0.24,0.08)	−0.38^***^ (0.47,-0.29)	(0.03,0.32)
Age(≤29 years)				
30–39 years	1.44^***^ (1.18,1.69)	−1.40^***^ (−2.04,-0.76)	1.81^***^ (1.59,2.04)	− 1.22^***^ (− 1.66,-0.77)
40–49 years	0.89^***^ (0.71,1.07)	−0.10 (− 0.44,0.23)	1.20^***^ (1.02,1.38)	− 0.73^***^ (− 1.00,-0.45)
50–59 years	0.47^***^ (0.30,0.64)	0.42^**^ (0.13,0.70)	0.76^***^ (0.59,0.92)	− 0.36^**^ (− 0.58,-0.13)
≥ 60 years	0.05 (− 0.12,0.23)	0.00 (− 0.28,0.28)	0.37^***^ (0.21,0.53)	−0.10 (− 0.31,0.10)
Marital status (Widowed)				
Never married	0.60^***^ (0.25,0.94)	0.89^**^ (0.33,1.45)	0.11 (−0.19,0.41)	0.56^*^ (0.12,1.00)
Married	0.35^**^ (0.10,0.60)	−0.47^**^ (− 0.80,-0.14)	0.21 (− 0.01,0.42)	−0.17 (− 0.44,0.10)
Divorced	0.08 (− 0.24,0.39)	0.08 (− 0.35,0.51)	−0.17 (− 0.56,0.23)	−0.13 (− 0.73,0.47)
Education (primary schools or below)				
Junior or senior high school	0.26^**^ (0.10,0.41)	−0.45^***^ (− 0.68,-0.23)	0.24^***^ (0.15,0.33)	− 0.56^***^ (− 0.72,-0.40)
Postsecondary and above	0.39^***^ (0.21,0.57)	− 1.27^***^ (− 1.60,-0.95)	0.43^*^ (0.04,0.82)	− 0.62 (− 1.52,0.27)
Father’s education (primary schools or below)				
Junior or senior high school	0.12^*^ (0.02,0.22)	− 0.15 (− 0.34,0.04)	0.14^*^ (0.02,0.26)	0.02 (− 0.20,0.25)
Postsecondary and above	− 0.01 (− 0.20,0.18)	−0.21 (− 0.62,0.21)	0.13 (− 0.62,0.88)	0.10 (− 1.22,1.43)
_cons	0.09(− 0.21,0.38)	−0.47^*^(− 0.87,-0.07)	0.14(− 0.09,0.38)	−0.38^**^(− 0.67,-0.09)
*N*	11,416		13,325	

^*^ *p* < 0.05, ^**^ *p* < 0.01, ^***^ *p* < 0.001

Factors significantly associated with health among urban residents included perceived air pollution, garbage pollution and noise pollution, gender, age, marital status, education, the father’s education; these were displayed in Model 1a and 1b. Model 1a showed that perceived air pollution had a negative effect on residents’ good health (B = -0.14, *p* < 0.05). Perceived noise pollution had a similar effect (B = -0.23, *p* < 0.001). Females were less likely to have good health than males (B = -0.38, *p* < 0.001). Residents aged 30–39, 40–49, 50-59 years were more likely to have good health than those aged 29 years or younger (B = 1.44, 0.89, 0.47, *p* < 0.001, respectively). Never married and married residents were more likely to have good health than widowed residents(B = 0.60, *p* < 0.001; B = 0.35, *p* < 0.01, respectively). Residents whose education level was primary school or below were less likely to have good health than those with a higher education. Residents whose father’s education level was junior or senior high school were more likely to have good health than those whose father’s education was primary school or below(B = 0.12, *p* < 0.05). Model1b showed perceived garbage pollution had a positive effect on residents’ poor health (B = 0.33, *p* < 0.01). Residents aged 30–39 years were less likely to have poor health (B = -1.40, *p* < 0.001) while residents aged 50–59 years were more likely to have poor health (B = 0.42, *p* < 0.01) than those aged 29 years or younger. Never married residents were more likely to have poor health (B = 0.89, *p* < 0.01) while married ones were less likely to have poor health (B = -0.47, *p* < 0.01) than widowed residents. Residents whose education level was primary school or below were more likely to have poor health than those with a higher education.

In addition, factors significantly associated with health among rural residents included gender, age, marital status, education level. Model 2a showed that females were less likely to have good health than males (B = -0.38, *p* < 0.001). Residents aged 29 years or younger were less likely to have good health than older ones. Residents whose education level was primary school or below were less likely to have good health than those whose education level was higher. Residents whose father’s education level was junior or senior high school were more likely to have good health than those whose father’s education was primary school or below (B = 0.14, *P* < 0.05). Model 2b showed residents aged 29 years or younger were more likely to have poor health than older ones. Never married residents were more likely to have poor health than widowed residents (B = 0.56, *p* < 0.05). Residents whose education level was junior or senior high school were less likely to have poor health than those whose education level was primary school or below (B = -0.56, *p* < 0.001).

## Discussion

This study was conducted to evaluate the impacts of perceived environmental pollution on health and analyzed the impacts of rural and urban residents’ perceived environmental pollution on health.

### Perceived environmental pollution

This study highlighted the impacts of perceived environmental pollution on health, especially among urban residents. The study found that the rates of urban residents who perceived air pollution (35.67%), water pollution (17.96%), garbage pollution (25.05%), and noise pollution (32.05%) were higher than those of their rural counterparts, which means that urban residents perceive more environmental pollution than their rural counterparts. This outcome was similar to the results of a local study on environmental awareness that used data from the *Chinese General Social Survey* (CGSS) in 2010 [21]. A Western study also found that urban residents are assumed to be more environmentally concerned than their rural counterparts [22]. Perceived air pollution, garbage pollution and noise pollution all had a significant negative impact on urban residents’ health, while none of the perceived pollution types had a significant impact on rural residents’ health. However, China’s environmental pollution is severe in both cities and villages. In recent years, the air pollution level has decreased quickly, although the levels are still high [23]. Despite measures such as limiting cars on the roads, reducing exhaust emissions and the technical development of air purification, China’s large-scale air pollution continues to spread from north to south. Some developed cities can use natural gas to better protect the environment, while less developed Chinese cities still choose coal and gas as fuel. Due its rapid spread, air pollution is quickly transmitted to neighborhood cities. This transmission has resulted in increasingly worse air pollution in both cities and villages in China. In rural China, the rates of stomach and liver cancer are 50% higher than those in the country’s major cities [27]. In part, this result may be due to the lack of water quality protection in rural areas. One of the goals published on November 8, 2018 by the Ministry of Ecology and the Environment and by the Ministry of Agriculture and Rural Affairs stated that by 2020, the utilization rate of fertilizers and pesticides in the country’s major crops will reach more than 40%, the coverage rate of soil testing and formula fertilization technology will reach more than 90%, the overall utilization rate of livestock and poultry fungi will exceed 75%, and the corresponding rate of equipment for sewage treatment plants in large farms will exceed 95% [30]. These numbers further emphasize that water pollution in Chinese rural areas has become a major hazard [31]. Moreover, garbage collection and treatment in rural areas has not been discussed until recent years [8]. Furthermore, little attention has been paid to noise pollution in rural areas in China. Relative to urban areas, environmental protection infrastructures, pollution supervision and environmental protection technologies in rural areas have greatly lagged behind. In addition, due to the pressure of strict environmental policies in cities, some heavily polluting enterprises have been transferred to the villages to weaken the risk of punishment from environmental protection departments and to reduce their penalty costs [25]. However, rural residents still lack awareness of the health risks of environmental pollution, partly due to inadequate policies aimed at controlling environmental pollution in rural areas. Disparate environmental health priorities in urban versus rural communities are also evident in the US [24], Canada [25], Japan [26], and Brazil [27]. There are two main reasons for those outcomes based on current research. First, differential healthcare access is highly related to disparities in health outcomes. Second, due to differences in their physical environments, rural residents may experience more exposure due to agricultural, forestry, or mining practices, while urban residents may experience more exposure due to traffic-related emissions, power generation, and industrial process [24]. The social environment is seldom considered an important factor influencing urban-rural differences in health outcomes, especially in Chinese research. In addition, health inequalities caused by environmental pollution may be broadened in the long term and are rarely discussed.

### Individual factors

The reported rate of good health among rural residents (68.08%) was slightly higher than that reported by their urban counterparts (66.64%). In addition, the reported rate of poor health among rural residents (8.69%) was higher than that among urban residents (7.14%).

### Gender

In this study, males were more likely to have a positive attitude towards their health status than females. These results are consistent with a study based on the first wave of the European Social Survey in 2003, which was conducted in 22 countries; in all countries, men report better health than women [28]. The study based on the CGSS in 2010 also presented similar results among both urban and rural residents in China [29]. Some studies have also found that women suffer from more nonfatal chronic conditions and a greater likelihood of functional limitations than men [30], although they live longer than men [31].

### Age

Residents aged 30 years or older were more likely to have a positive attitude towards their health status than were younger respondents, which applied to both urban and rural residents. Although health status declined with an advanced age [29] and young people were reported being more energetic and exercising more [32, 33], a greater socioeconomic status (SES) will highly promote residents to take advantage of new mechanisms that protect and promote better health and reduce their mortality risks [34]. Young residents aged 29 years or younger are more likely to have lower SES levels compared with older residents.

### Education level

Residents whose education level was primary school or below were more likely to have a negative attitude towards health than were those with a higher education level among both urban and rural residents, which is similar to the results of local studies showing that years of schooling have a positive impact on residents’ self-rated health [29, 35]. Education level is an indicator of SES and is correlated with income and occupation, as well as working and living conditions [36]. Residents with low education levels are more likely to be influenced by disease and to have no access to medical interventions. A Western study also found that a higher level of education is positively associated with longer life and better health throughout the lifespan [37].

### Marital status

Most studies have shown that married adults enjoy better physical and mental health than unmarried adults [38] and have longer life expectancies than [39] divorced adults. In this study, compared with widowed residents, never married residents were more likely to be in good or poor health when comparing general health outcomes. In a universal marriage system, the tradition of marriage coexists with the modern culture of being single in China. Young residents enjoying their single status may have good health; however, others who did not get married at a marriageable age may have poor health when compared with individuals who were ever married, even if they lost their spouse. These widowed individuals are viewed as more successful in society based on the marriage tradition. Additionally, married or ever married residents were more likely to have a positive attitude towards their health than were others [29].

### Family factors

In this study, the father’s education level had a positive impact on urban and rural residents’ good health. The father’s education level is an important factor with regard to the family’s SES. A higher paternal education level means that the family’s income and occupation may be better, which creates better nutrition during the residents’ childhood and more medical resources when they face certain health risks.

### Implications

The study has several implications. First, more advocates for environmental pollution should be provided, especially for rural residents, and these advocates should make the residents take notice of the seriousness of the air, water, garbage and noise pollution. This advocacy would help residents build an awareness of environmental protection in their daily life. Second, more official reports on the impact of environmental pollution on health in China should be published. The State of the Environment Report annually published by the Ministry of Ecology and Environment of China plays an important role in acknowledging the developments made by the Chinese government in enhancing the environment. However, little attention has been paid to rural residents and their environmental awareness. Third, measures to increase the protection of rural environments should be taken. For instance, some highly polluting enterprises should not be allowed to produce in villages. These enterprises continue to transfer pollution to rural areas, which adds risks and vulnerability to rural environments. This result may create risks for agricultural production and cause the peasants to lose their livelihood. More research is needed to determine the factors that influence rural residents’ health and their environmental awareness, as environmental right inequalities may create more health inequalities between urban and rural residents.

### Limitations

First, as a cross-sectional study, this study solely exhibits a static picture that cannot thoroughly describe the changes between the actual impacts of perceived environmental pollution on residents’ health. The questionnaire did not cover the participants’ demand to know about environmental pollution and health risks, which was relevant to the policy implications of the study. Second, the study captured some demographic and environmental factors in residents’ health but could not take all the individual differences into consideration, such as nutrition and physical exercise. Third, the results of this study were based on measurements of self-rated health. This approach may not reflect the real status of the residents’ physical health. In following studies, some objective assessments need to be added, such as those pertaining to chronic diseases. Finally, the large differences in the awareness of environmental pollution’s impacts on health between urban and rural residents are partly due to the priority of environmental protection policies in cities. This study did not involve the impacts of environmental policies.

## Conclusions

Rural residents perceive little about the impacts of environmental pollution on health, which may create risks and vulnerabilities of rural environments and the livelihood of these residents. Suitable measures should be executed to more readily accomplish the supportable improvement of urban and rural regions together. Great attention should be paid to the impacts of environmental pollution on health for not only urban residents but also rural residents, which will highly improve the support of green development among the public in China.

## Supplementary information

**Additional file 1.** Questionnaire

## Data Availability

The datasets used and/or analyzed in the current study cannot be publicly shared and they are available from the corresponding author on reasonable request.

## Abbreviations

ACWF: All China Women’s Federation
CGSS: Chinese General Social Survey
SES: Socioeconomic status
SSCW3: The 3rd Survey on the Status of Chinese Women in 2010
