# Supplementary material for: Association between perceived environmental pollution and health among urban and rural residents-a Chinese national study
Source: BMC Public Health. 2020 Feb 6;20:194. doi: 10.1186/s12889-020-8204-0 (PMC7006119; doi:10.1186/s12889-020-8204-0)
Supplement: Supplementary file 1 — Additional file 1. Questionnaire [file 12889_2020_8204_MOESM1_ESM.docx]

The 3^rd^ Survey on the Status of Chinese Women in 2010

Personal questionnaire

Hello! I am an investigator commissioned by the All China Women's Federation and the National Bureau of Statistics, and I am conducting a survey on the social status of women in our country. This survey is of great significance for the party and the state to formulate relevant social policies. Based on random sampling, you were selected for survey. As long as your answer matches your real thoughts and actual situation, it doesn't matter whether it is right or wrong. We will keep your personal and family information confidential in strict accordance with the relevant provisions of the law. The investigation will take you some time, and I hope to be supported. Thank you!

the All China Women's Federation and the National Bureau of Statistics

Dec 2010

〖Starting time:▁▁Month▁▁Day▁▁Hour▁▁Minute〗

**Please tell me some basic information about you**:

A1 Gender: ⬜

1 Male 2 Female

A2 Birth date: Month Day Year

A3 Education level: ⬜

1 Illiterate 2 Primary school 3 Junior high school

4 Senior high school 5 Technical secondary school/Technical college 6 Postsecondary 7 Undergraduate 8 Postgraduate 9 Others (please specify) **_______**

A4 Marital status: ⬜

1 Unmarried 2 Married 3 Divorced 4 Widowed 8 Unclear

A5 Hukou status: ⬜

1 Urban 2 Rural 3 None 8 Unclear

A6 The father’s education level: ⬜

1 Illiterate or illiterate 2 Primary school 3 Junior high school

4 Senior high school 5 Technical secondary school/Technical college 6 Postsecondary 7 Undergraduate 8 Postgraduate 9 Unclear

A7 Did you perceive the following environmental pollution in your daily life?

|  | No | Yes | unclear | A7 | |
| --- | --- | --- | --- | --- | --- |
| A Air pollution | 0 | 1 | 8 | A |  |
| B Water pollution | 0 | 1 | 8 | B |  |
| C Garbage pollution | 0 | 1 | 8 | C |  |
| D Noise pollution | 0 | 1 | 8 | D |  |

A8 Overall, how do you feel about your current health? ⬜

1 Very good 2 Good 3 Fair 4 Poor 5 Very poor 8 Unclear

Could you please leave your phone number so that we can contact you if needed.

Respondent's Phone number:

〖Ending time:▁▁Month▁▁Day▁▁Hour▁▁Minute〗
